# Supplementary material for: Chromothripsis during telomere crisis is independent of NHEJ, and consistent with a replicative origin
Source: Genome Res. 2019 May;29(5):737–49. doi: 10.1101/gr.240705.118 (PMC6499312; doi:10.1101/gr.240705.118)
Supplement: Supplemental Material [file supp_gr.240705.118_Supplemental_file_1.zip › contigs/annotated_contigs/DB114/contig.2.DB114_length_661_mean_cov_4.99243570348.docx]

**DB114_length_661_mean_cov_4.99243570348**

```
TCATTGTATCCTAATATACAAATATATTAGAATTAATTTGATCTATTCTGCCACCAATGGACATTGGATTATATCCAGTTTGGGGCAAT
 >chr2:127393850-127394187 - E=5e-191 p=8e-03
TATAAGTAATGTTGCTATGAAAATTCTTGTGCATGTACATGTACCCTGGTGTTCAGGTACACACATTTCTGTTGGATATGTATTTACAC

AGATATATACACACATGCACACACTATACATTATATATACATACTATATATAATATATGTGCATACTATACATATTATGTATACACATT

ATATACATAATATATACAGATACTACACATACTCTATGTATAGACACATACACAATGTACATA|ATATACA|TATATATATATATAGAC
                                                                >chr2:127392988-1273933
TGGGGAATAGTAGGTGCCCAATAAGTGGTGACTGTTTTTACCTCGTATCTTCACTTATTAGTGGTAAAGTCACCAATGGAAACAAAGCA
19 - E=2e-178
GACAGAGTTCCAGCAGACTGTGAGGAAGCAGCAGGAGTCTTGAGGGTTCACTGCTGAATGGTCCCCTGGGATAGCTTGTACTGTTGTGC

AACAAATATTTACTCTTCCCTTCCCCAGCATGAGCCCAGTGCACTGCCTTACCCTAGTCCTCTGGGATTTGCTTTGGCTGGGGGAATGT

GAGTGAACATGACCTGAGTGTATCCCAGCACAGTTTGGAA
```
